# Persistent Infections with Diverse Co-Circulating Astroviruses in Pediatric Oncology Patients, Memphis, Tennessee, USA 

**DOI:** 10.3201/eid2302.161436

**Published:** 2017-02

**Authors:** Valerie Cortez, Pamela Freiden, Zhengming Gu, Elisabeth Adderson, Randall Hayden, Stacey Schultz-Cherry

**Affiliations:** St. Jude Children’s Research Hospital, Memphis, Tennessee, USA

**Keywords:** astrovirus, human astroviruses, immunocompromised host, virus shedding, gastroenteritis, genotype, prevalence, viruses, Tennessee, United States, children, pediatric, enteric infections, HAstV, co-circulation, cancer, oncology

## Abstract

Human astroviruses are a major cause of pediatric gastroenteritis, especially in immunocompromised children. We conducted a retrospective study to demonstrate that diverse astrovirus genotypes can co-circulate in pediatric oncology patients. A subset of cases is associated with long-term virus shedding (range 17–183 days).

Astroviruses are a leading cause of diarrhea, and children <2 years of age and immunocompromised persons are at higher risk for systemic and severe disease (*1*). Few studies have investigated the diversity of astroviruses that infect these populations despite there being 3 distinct phylogenetic clades of human astroviruses (HAstVs) (canonical genotypes HAstV1–8 and noncanonical genotypes MLB1–3 and VA1–5), which makes surveillance challenging (*2*). To explore the genetic diversity of astroviruses in persons with high-risk for infection, we performed a retrospective study in immunocompromised pediatric oncology patients, analyzing remnant fecal samples collected in 2008 and 2010–2011.

## The Study

A total of 909 remnant fecal samples were collected from 419 patients; 473 samples from 220 patients were collected during January–December 2008, and 436 samples from 199 patients were collected during January 2010–June 2011 (Technical Appendix Figure 1). All samples were de-identified, so that only a patient identification code, sample identification code, and date were known. No other clinical data were available. The St. Jude Children’s Research Hospital Institutional Review Board approved this study with a waiver of consent.

We extracted viral RNA from homogenized fecal samples by using the MagMAX-96 Viral RNA Isolation Kit (Applied Biosystems Life Technologies, Carlsbad, CA, USA). We screened samples from 2008 by using a singleplex real-time reverse transcription PCR (rRT-PCR) (*3*), an in-house multiplex PCR to identify canonical (HAstV1–8) and noncanonical (MLB1 and VA2) genotypes (*4*), and endpoint PCRs by using primers targeting open reading frame (ORF) 1(*5*) and ORF2 (*6*,*7*). The singeplex method detected HAstV in 23/72 samples, compared with 61/67 (5 samples were not tested) by the multiplex method. We screened samples from 2010–2011 solely by the multiplex method; 14 were positive for HAstV. Overall, we detected HAstV in 86 (9.5%) of 909 samples from 60 (14.3%) of 419 patients (Table; Technical Appendix Table), giving a detection rate comparable to those reported for other immunocompromised populations (*8*,*9*).

**Table:** Characteristics of HAstV-positive fecal samples from pediatric oncology patients, Memphis, Tennessee, USA, 2010–2011*

Patient ID	Sample ID	Enteric co-infection†	Genotype‡
SJ1§	4	ND	HAstV2
	8	ND	HAstV1
SJ2	9	ND	VA2
SJ3	10	Norovirus	UND
SJ4	5	ND	UND
SJ5	11	ND	VA2
	12	ND	VA2
SJ6	14	ND	UND
SJ7	13	ND	UND
SJ8	1	ND	HAstV5
	2	ND	HAstV5
SJ9	6	Norovirus	HAstV1
SJ10	3	ND	HAstV1
SJ11	7	ND	MLB1

*HAstV, human astrovirus; ID, identification; ND, not detected; UND, undetermined; rRT-PCR, real-time reverse transcription PCR. †Samples positive for either norovirus or sapovirus by rRT-PCR. ‡Genotype determined by partial open reading frame 1b and 2 sequencing. §Patient had sequential infections with different genotypes.

**Figure 1:**
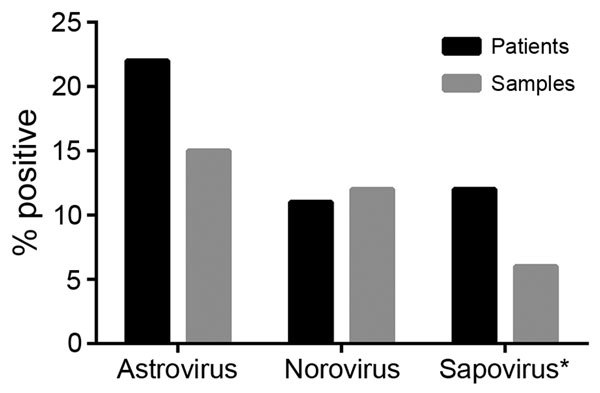
Enteric virus infections identified from remnant fecal samples from pediatric patients with cancer, Memphis, Tennessee, USA, 2008. The percentage of samples and patients testing positive for human astrovirus was higher than the percentages testing positive for either norovirus or sapovirus. *Due to limited sample availability, 31 samples could not be tested for sapovirus.

To place these findings in the context of those for other enteric virus infections, we also used rRT-PCR methods to determine the prevalence of norovirus and sapovirus in the fecal samples from 2008 (*10*,*11*). Due to sample quantity limitations, we were unable to test 31 samples for sapovirus. We detected HAstV in the highest proportion of patients and samples (22% [49/220] and 15% [72/473], respectively), followed by norovirus (11% [39/220] and 12% [58/473]) and sapovirus (12% [25/211] and 6.6% [29/442]) (Figure 1). Together, these data demonstrate that HAstVs are a major contributor to the enteric virus infections in this patient population. Furthermore, co-infection occurred in only 16% of HAstV-positive patients (Table; Technical Appendix Table), much lower than the 33%–65% of patients reported in other studies (*2*).

A median of 40 (range 18–54) samples were collected each month in 2008; cases peaked in spring and decreased in summer and early fall (Technical Appendix Figure 2). We further investigated temporal trends in HAstV infections by using longitudinal samples from 34 patients (2–12 samples/patient) collected over a median of 81 (range 3–328) days. Of the 34 patients, 12 were previously HAstV negative, indicating that more than one third of patients had newly acquired infections (Technical Appendix Table). Of the 12 patients with >1 positive specimen, 6 experienced prolonged HAstV shedding (defined by positive samples collected >2 weeks apart [range 17–183 days]) (Technical Appendix Table). Subsequent fecal samples for 2 patients (SJ35 and SJ209) were negative for HAstV, suggesting that the virus had cleared, whereas the other 4 patients (SJ22, SJ175, SJ245, and SJ275) had detectable HAstV in their final fecal samples. Despite being co-infected with HAstV and norovirus, patient SJ275 persistently shed only HAstV, highlighting the need to further explore enteric virus co-infections and the potential for virus interference.

**Figure 2:**
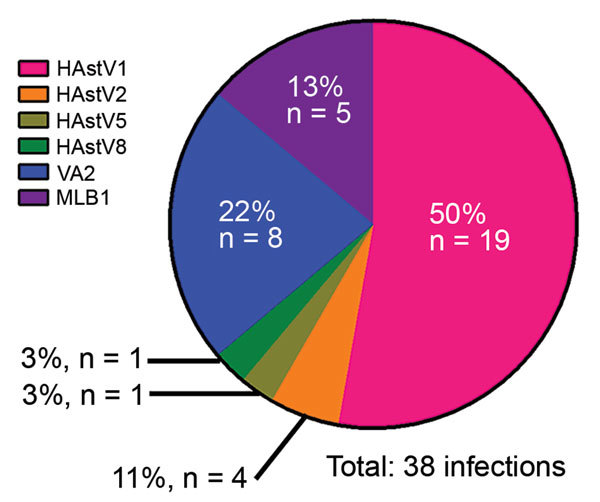
Co-circulating human astrovirus (HAstV) strains in pediatric patients with cancer in Memphis, Tennessee, USA. Six different HAstV genotypes were identified among the HAstV-positive fecal samples collected from pediatric patients in 2008 or in 2010–2011; more than one third of the viruses were noncanonical VA and MLB genotypes.

We genotyped 50 of the 86 HAstV-positive samples by using the aforementioned endpoint PCR methods, with patient-specific primers and the 3′ RACE System (Invitrogen Life Technologies, Carlsbad, CA, USA) for rapid amplification of cDNA ends to obtain partial open reading frame (ORF) 1b (RNA-dependent RNA polymerase) and ORF2 (capsid protein) sequences. We were unable to genotype the remaining positive samples because of inadequate sample quality or quantity. We used 50 sequences, collectively representing 38 unique infections, in the phylogenetic analysis (Technical Appendix Figure 3) and BLAST (https://blast.ncbi.nlm.nih.gov) searches for samples with only shorter sequence reads available. Tree topology of ORF1b and ORF2 sequences indicated that none of the samples contained recombinant viruses or was co-infected with different genotypes. Overall, HAstV1 was the most prevalent genotype identified (n = 19, 50%), followed by the noncanonical genotypes VA2 (n = 8, 21%) and MLB1 (n = 5, 13%) (Figure 2). Fecal samples from 4 (11%) patients were positive for HAstV2; 1 each was positive for HAstV5 and HAstV8. Of note, 4 patients were sequentially infected with >1 genotype, and 3 patients from 2008 had serial infections with canonical and noncanonical genotypes (Table; Technical Appendix Table). The average interval between these infections was ≈7 months.

## Conclusions

In a previous study of immunocompromised children hospitalized with diarrhea, the prevalence of HAstV infection was 5% (*9*). HAstV detection in HIV-infected persons of all ages with and without gastroenteritis has been reported to be as high as 12% (*8*). Thus, the 14% detection rate we observed in pediatric oncology patients is consistent with the rate in previous reports. Some patients showed prolonged virus shedding, which has been reported in immunocompetent and immunocompromised children, in some cases for as long as 3 months (*12*,*13*). However, 4 of the patients in our study shed virus beyond 3 months, including 1 who shed for ≈6 months, highlighting the ability of HAstV infections to persist within this population. In addition, 3 of the 4 patients with prolonged shedding were infected with the noncanonical genotypes MLB1 or VA2. Although genotypic differences in virus load and long-term shedding have been reported for canonical viruses (HAstV1–8) (*12*), we are not aware of such studies with noncanonical viruses. A larger, prospectively followed, longitudinal cohort would be required to investigate these differences.

Our identification of 4 patients with sequential HAstV infections is notable, especially because all cases occurred during 1 year of observation. One previous report also described a child who was first infected with HAstV3 and then, 9 months later, with HAstV1 (*14*). Although our study showed more than one third of the HAstV infections detected in samples from 2008 were newly acquired, we do not know the precise circumstances of these infections—whether they were associated with healthcare settings, or if they occurred at particular times during the patients’ treatment. The trend of HAstV infections in our study appears to mirror that in reports from Egypt, Brazil, and the eastern United States, which also observed a peak of infections in late spring and early summer (*13*).

In summary, we used multiple molecular methods to identify HAstV infections in immunocompromised pediatric oncology patients, enabling us to examine a broad representation of the infections experienced by this population and to identify 6 co-circulating viruses. We showed that the singleplex rRT-PCR method (*3*) was unable to capture infections caused by noncanonical viruses and is, therefore, limited in its utility for accurate surveillance and diagnosis. Although our multiplex method was able to identify all canonical and 2 noncanonical viruses (*4*), it is possible that we missed infections with other strains. Given the limited knowledge about HAstV genotypes and clinical disease, particularly in immunocompromised persons, additional studies will be crucial to help develop better diagnostics to capture all known HAstV genotypes.

Technical Appendix Characteristics of human astrovirus (HAstV)–positive samples, pipeline for HAstV detection, temporal distribution of HAstV and phylogenetic trees of open reading frame 1b and 2 sequences.
