# Supplementary material for: Persistent Infections with Diverse Co-Circulating Astroviruses in Pediatric Oncology Patients, Memphis, Tennessee, USA
Source: Emerg Infect Dis. 2017 Feb;23(2):288–90. doi: 10.3201/eid2302.161436 (PMC5324824; doi:10.3201/eid2302.161436)
Supplement: Technical Appendix — Characteristics of human astrovirus (HAstV)–positive samples, pipeline for HAstV detection, temporal distribution of HAstV and phylogenetic trees of open reading frame 1b and 2 sequences. [file 16-1436-Techapp-s1.pdf]

Article DOI: http://dx.doi.org/10.3201/eid2302.161436

Persistent Infections with Diverse Co-Circulating Astroviruses in Pediatric Oncology Patients, Memphis, Tennessee, USA

Technical Appendix

Technical Appendix Table. Characteristics of HAstV-positive fecal samples collected from pediatric oncology patients, Memphis, Tennessee, USA, 2008*

Patient ID	Sample ID	rRT-PCR detection method†		Enteric co-infections‡	No. time points positive/no. total	New infection§	Virus shedding, days	Genotype¶
		Singleplex	Multiplex					
SJ22	166	–	+	ND	4/4	No		UND
	193	–	+	ND		No		VA2
	251	–	+	ND		No		VA2
	266	–	+	ND		No	64	VA2
SJ35	253	–	+	ND	2/5	No		UND
	272	–	+	ND		No	17	UND
SJ48	215	–	+	ND	2/7	Yes		HAstV1
	226	+	–	ND		No		HAstV1
SJ54	225	+	+	ND	1/1	No		HAstV1
SJ60	212	+	+	ND	1/1	No		HAstV8
SJ105	234	–	+	ND	1/2	No		UND
SJ109	210	–	+	ND	1/1	No		UND
SJ114	508	–	+	ND	3/3	No		MLB1
	516	–	+	ND		No		MLB1
	518	–	+	ND		No		MLB1
SJ116	347	–	+	ND	1/1	No		UND
SJ122	124	+	NT	Norovirus	1/2	Yes		HAstV2
SJ127	259	+	+	ND	1/1	No		HAstV1
SJ133#	214	–	+	ND	4/4	No		MLB1
	304	–	+	ND		No		UND
	566	+	+	ND		No		HAstV1
SJ135#	276	–	+	ND		Yes		VA2
	561	–	+	ND		No		MLB1
SJ145	184	–	+	ND	2/6	Yes		HAstV1
	186	–	+	ND		No		UND
SJ149	125	+	NT	ND	1/1	No		HAstV2
SJ150	183	–	+	ND	1/4	Yes		UND
SJ162	237	–	+	ND	1/2	No		UND
SJ163	221	+	+	ND	1/2	Yes		HAstV1
SJ164	211	+	+	ND	2/6	No		HAstV1
	232	–	+	ND		No		UND
SJ175#	301	–	+	ND	4/5	Yes		HAstV1
	509	–	+	ND		No		VA2
	525	–	+	ND		No		VA2
	555	–	+	ND		No	24	VA2
SJ176	261	+	–	ND	1/4	Yes		HAstV1
SJ177	110	+	NT	ND	1/1	No		HAstV2
SJ179	107	+	–	Sapovirus	1/2	No		HAstV1
SJ186	305	–	+	ND	1/1	No		UND
SJ190	122	+	NT	Norovirus	1/1	No		UND
SJ191	182	–	+	ND	1/3	No		VA2
SJ195	123	+	NT	ND	1/3	No		UND
SJ201	263	–	+	ND	1/5	Yes		UND
SJ203	246	–	+	ND	2/3	Yes		UND

Patient ID	Sample ID	rRT-PCR detection method†		Enteric co-infections‡	No. time points positive/no. total	New infection§	Virus shedding, days	Genotype¶
		Singleplex	Multiplex					
	248	–	+	ND		No		HAstV1
SJ207	278	–	+	ND	1/1	No		UND
SJ208	252	–	+	Sapovirus	1/2	No		VA2
SJ209	213	+	+	ND	3/6	No		UND
	233	–	+	ND		No		UND
	307	–	+	ND		No	95	UND
SJ210	308	–	+	ND	1/2	No		UND
SJ215	299	–	+	Norovirus	1/12	Yes		UND
SJ216	227	+	+	ND	1/3	No		HAstV1
SJ217	249	–	+	ND	1/6	No		HAstV1
SJ218	268	–	+	ND	1/1	No		UND
SJ223	258	–	+	ND	1/1	No		UND
SJ228	306	–	+	Norovirus	1/1	No		UND
SJ231	269	–	+	ND	1/1	No		UND
SJ233	280	–	+	ND	1/8	No		UND
SJ241	467	–	+	Norovirus	1/5	Yes		UND
SJ245	309	–	+	ND	4/6	No		UND
	536	–	+	ND		No		MLB1
	551	–	+	ND		No		MLB1
	573	–	+	ND		No	183	MLB1
SJ252	546	–	+	ND	1/2	Yes		VA2
SJ271	575	+	+	ND	1/4	Yes		UND
SJ275	387	+	+	Norovirus	3/7	No		UND
	576	+	+	ND		No		HAstV1
	580	+	+	ND		No	129	HAstV1
SJ285	417	+	–	ND	1/4	No		UND
SJ291	578	+	+	ND	1/1	No		HAstV1
SJ298	583	+	–	ND	1/2	Yes		HAstV1

*HAstV, human astrovirus; ID, identification; ND, not detected; NT, not tested; UND, undetermined; rRT-PCR, real-time reverse transcription PCR; +, positive; –, negative.
†rRT-PCR methods used as described (main text citations): singleplex (*3*); multiplex (*4*).
‡Samples positive for either norovirus or sapovirus by rRT-PCR.
§Evidence of new infection indicated if ≥1 preceding sample tested negative by rRT-PCR.
¶Genotype determined by partial open reading frame 1b and 2 sequencing.
#Patients who had sequential infections with different genotypes.

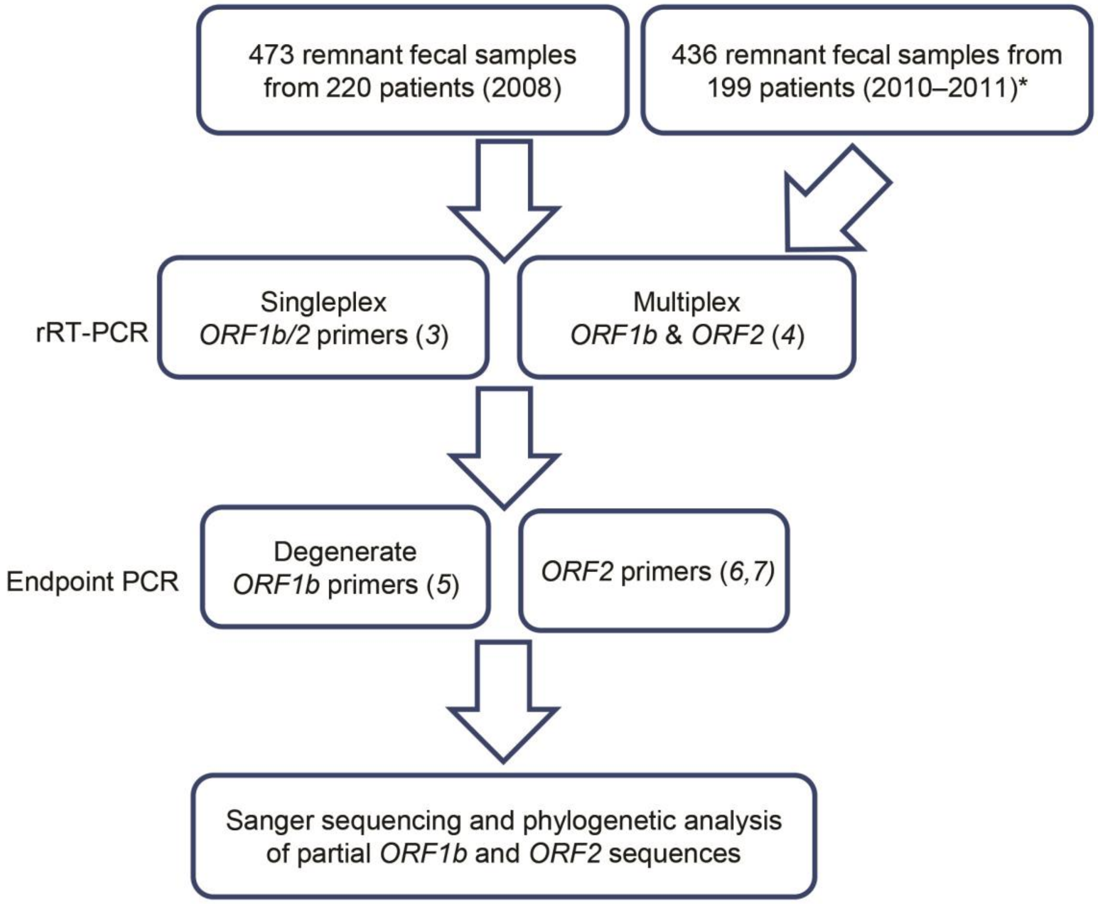

Technical Appendix Figure 1. Pipeline for detecting human astroviruses by independent real-time reverse transcription PCR methods and endpoint PCR. Two groups of remnant fecal samples from 2008 and 2010–2011 were examined. Samples from 2010–2011 were previously screened by the multiplex rRTvPCR method in the main text reference (*4*). All samples were submitted to the clinical diagnostic laboratory at St. Jude Children’s Research Hospital (Memphis, Tennessee, USA). ORF, open reading frame.

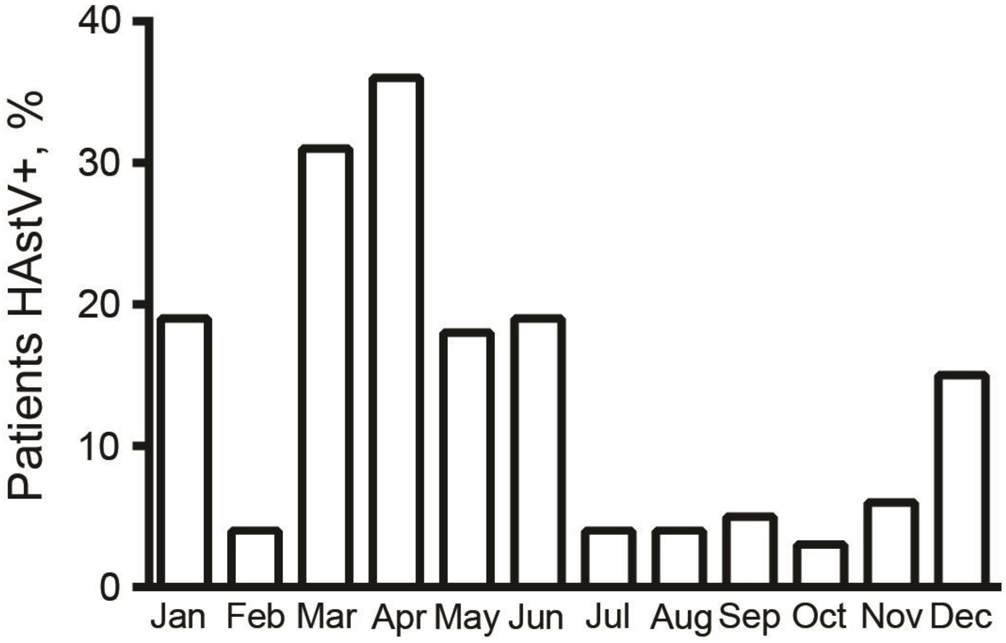

Technical Appendix Figure 2. Monthly distribution of human astrovirus (HAstV) detection in fecal samples collected from patients at St. Jude Children's Research Hospital (Memphis, Tennessee, USA) in 2008. HAstV was detected in fecal samples from patients in every month, with the peak period of detection beginning in March and lasting until June.

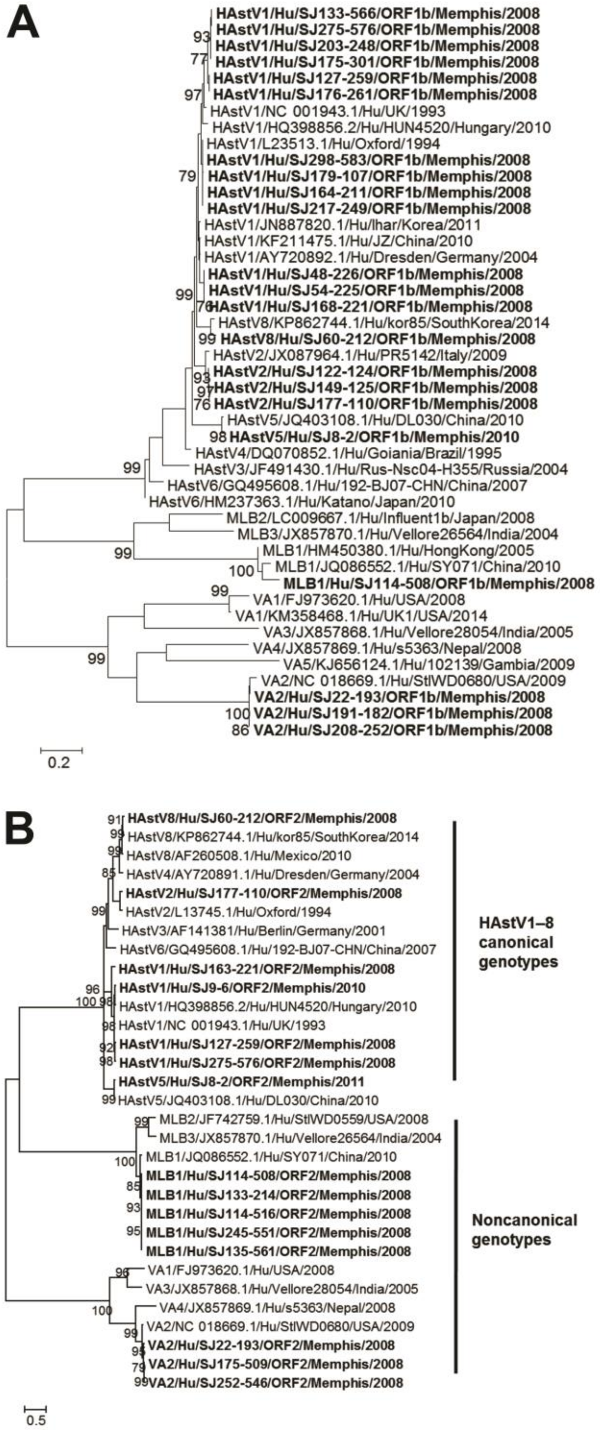

Technical Appendix Figure 3. Phylogenetic analysis of partial ORF1b and ORF2 sequences to identify canonical and noncanonical human astrovirus (HAstV) genotypes. Amplicons (380 bp) for ORF1b (A), corresponding to residues 835 to 1203 (HAstV1 GenBank accession no. L23513.1), and the first 1000 bp of ORF2 (B) were aligned with reference sequences using MUSCLE (MEGA6, version 6.0). Phylogenetic relationships were inferred by maximum likelihood estimation (MLE) using a general time-reversible

model with a discrete gamma distribution. Construction of phylogenetic trees was based on 1,000 bootstrapped replicates. The branch lengths represent the number of substitutions per site, and bootstrap values greater than 70 are shown. Reference sequences, including their GenBank accession numbers, are noted in the tree. Sequences from pediatric patient samples from St. Jude Children's Research Hospital (Memphis, Tennessee, USA) are in bold and year collected is noted (GenBank accession nos. KX932047–932067). ORF, open reading frame.
